# A Case of Disseminated Lyme Disease Presenting as Chronic Urticaria

**DOI:** 10.7759/cureus.5600

**Published:** 2019-09-09

**Authors:** Mary Michael, Tamara Gmitter

**Affiliations:** 1 Dermatology, Lake Erie College of Osteopathic Medicine, Tampa, USA; 2 Dermatology, Florida Atlantic University School of Medicine, Boca Raton, USA

**Keywords:** lyme disease, urticaria, erythema migrans

## Abstract

We present a case of cyclical bouts of chronic urticaria on the abdomen, scalp, and hands in a patient with disseminated Lyme disease. Lyme disease traditionally presents as a cutaneous lesion known as erythema migrans, which alerts patients and physicians that diagnosis and treatment is necessary. This case highlights the importance of variable cutaneous presentations of Lyme disease, especially if nerve pain is concurrent.

## Introduction

Lyme disease is caused by the *Borrelia burgdorferi* spirochete transmitted to the human host after a bite usually from the *Ixodes* deer tick [1]. Although primarily a disease of the Northeast United States, it is rapidly spreading to the Southern states as well; this should be kept in mind with early detection to increase the chance of a full recovery [2]. We present a patient with recurring dermatitis for five years. A workup revealed chronic Lyme disease. This case highlights the importance of considering Lyme disease when a patient presents with unusual dermatologic lesions and unconventional symptoms. The patient was misdiagnosed with urticaria before presenting to our office, an oversight which potentially affected his care and prognosis.

## Case presentation

**Figure 1:**
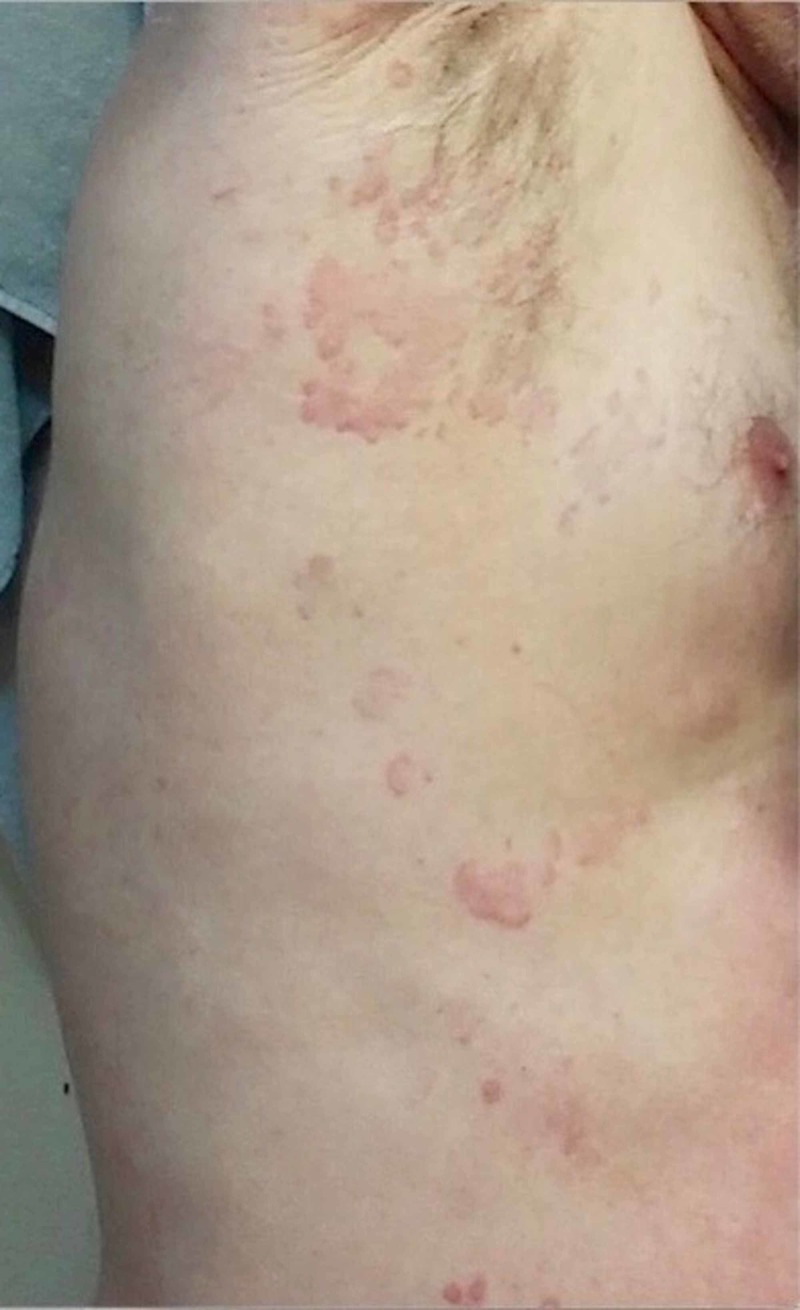
Oval, pink, and serpiginous plaques over the flank and abdomen

A 49-year-old male, who works as an outdoor city inspector in South Florida, presented to the dermatologist with a history of a chronic, idiopathic rash. Since the rash was not present during the current visit, the patient brought photos on his phone of breakouts in the past. One photo showed erythematous eruption of multiple oval, pink plaques serpiginous over the flanks and abdomen (Figure 1).

**Figure 2:**
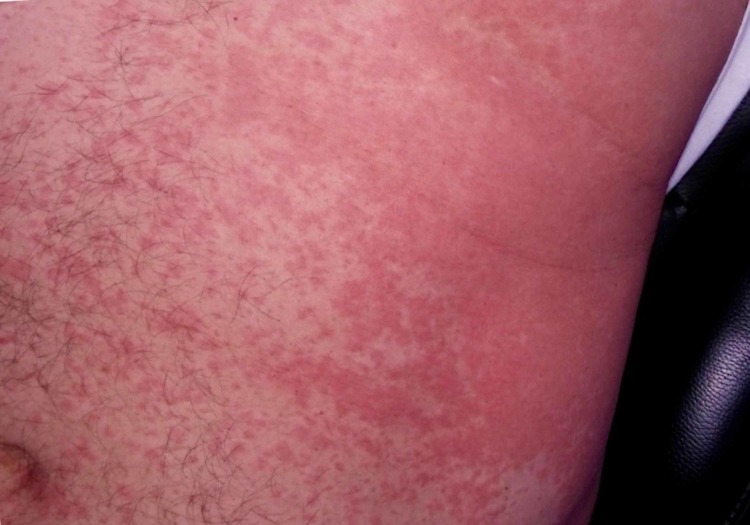
A large plaque covering the entire abdomen and flank with diffuse, coalescing papules

Another photo showed a large plaque covering the entire abdomen and flank with diffuse, coalescing papules (Figure 2).

**Figure 3:**
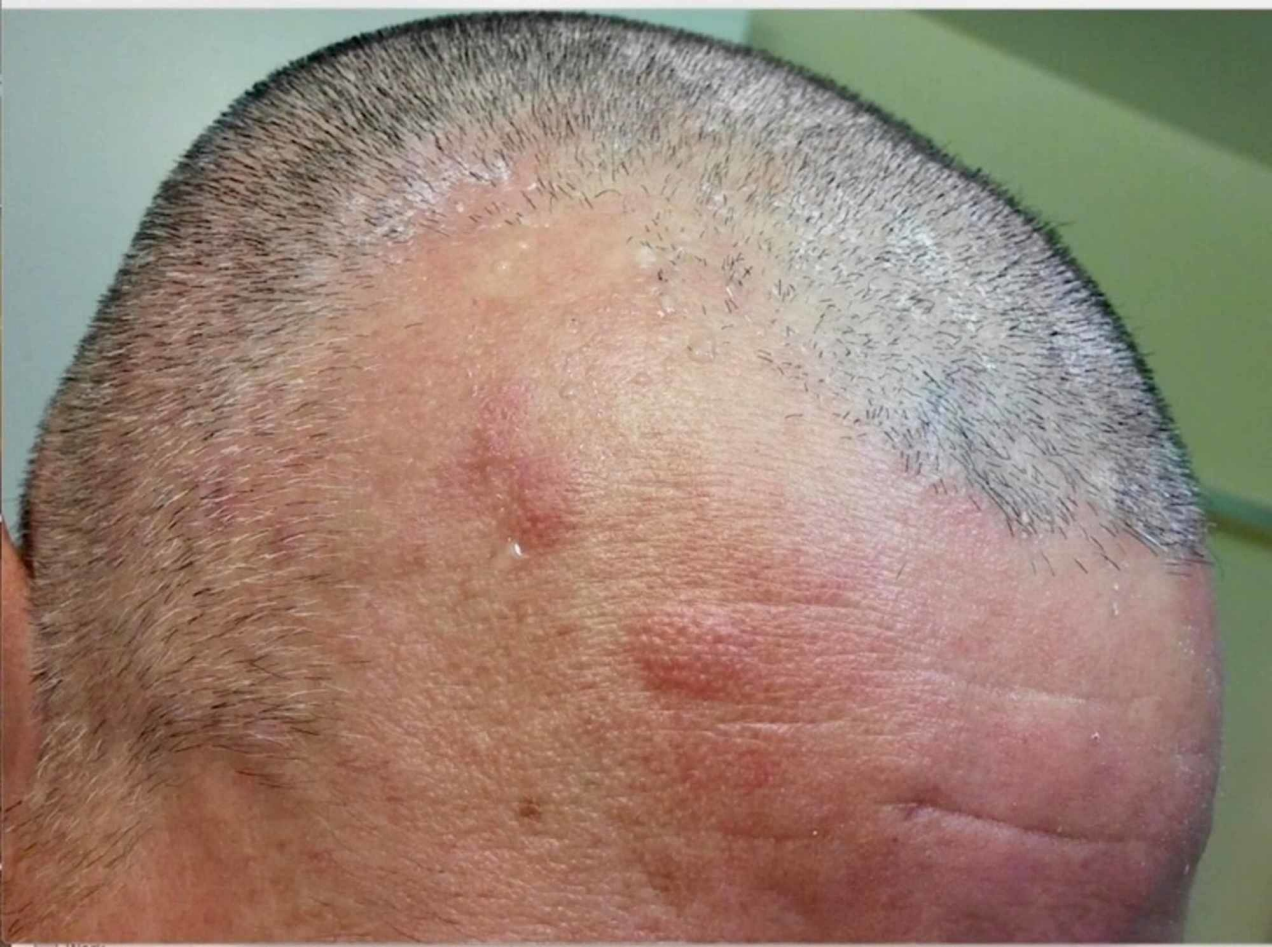
Non-scaly, oval, and pink plaques over scalp

He also had well-demarcated, 1 cm to 2 cm, non-scaly, oval, and pink plaques over the bilateral hands and scalp (Figure 3).

He reported having similar lesions for the past three years, at least twice a year and six months apart that resolved on their own after two weeks. During one episode in 2016, the patient had a biopsy of the forehead that demonstrated urticaria. Over the past three years, the patient had visited multiple dermatologists, primary care physicians, and urgent care clinics and was treated with cortisone, which brought temporary resolution of the lesions. Rheumatology work up in the past was negative. One week prior to his visit to our office, he received intramuscular Kenalog (Bristol-Myers Squibb, Princeton, NJ) in an urgent care clinic with improvement of symptoms and rash. He denied a link with food or drugs and had no known allergies. On physical exam, the patient had a resolving eruption with very faint erythema over the abdomen and left flank. Due to the previous diagnosis of urticaria and resolution of the lesions, the patient was referred to an allergist for further work up. 

The patient returned two days later. The dorsum of bilateral hands had erythematous, tender patches over the knuckles and an oval, 0.7 cm diameter macule on the dorsum of the mid-right wrist. The remainder of the body was clear of lesions. He complained of sharp, pruritic, and burning neuropathy over the bilateral hands, feet, and head. He reported worsening of the symptoms with cold water, yet warm water lessened the pain. Due to his symptoms, laboratory blood work consisting of a complete metabolic panel, complete blood count (CBC) with differential, platelets, Lyme disease antibodies with Western blot, heavy metal, and arsenic panel, rapid plasma reagin (RPR), and connective tissue work up was ordered which included anti-nuclear antibody (ANA) titer, double-stranded DNA (dsDNA), Smith antibody, Sjögren's syndrome-related antigen (SSA) antibody, C-reactive protein, and Anti PM/SCL 100AB. Abnormal results included a positive titer for Lyme disease with eight out of ten of the criteria for immunoglobulin G (IgG) positivity. The immunoglobulin M (IgM) blot was negative. The patient was diagnosed with Lyme disease and referred to an infectious disease physician, who prescribed doxycycline for two weeks. Repeat labs were still positive for the antigens. The patient was placed on daily intravenous (IV) ceftriaxone for 45 days with repeat labs planned for the future.

## Discussion

Following a bite from the *Ixodes* tick, the course of Lyme disease, caused by the spirochete *Borrelia burgdorferi, *typically progresses through three stages. Stage one, also known as the localized disease, includes erythema migrans (EM), an erythematous rash that spreads centrifugally from the site of the bite. Along with the rash, flu-like symptoms develop. The second stage, known as the early dissemination period, spirochete dissemination, which primarily targets the cardiac and neurological systems. This occurs weeks to months after the tick bite. The third stage or late dissemination period causes rheumatologic disease and more severe neurological disease [1]. Patients seek medical attention once clinical symptoms become apparent, most likely EM or flu-like symptoms. Up to 80% of patients experience some type of dermatologic condition, but only 19% experience the classic “bulls-eye” rash or EM [3]. This means that a substantial number of patients or more are presenting with other manifestations of Lyme disease with the possibility of being untreated for long periods of time.

Between 2006 and 2016, there was a 31.47% increase of confirmed Lyme disease in the United States [2]. Risk of exposure to the infected ticks increases with certain recreational or occupational activities such as time outdoors during summer months in grasslands [1]. Each year the spirochete-infested ticks spread farther from their primary foci of the Northeast and the upper Midwest of the United States. Due to this, physicians all over the country need to have a low threshold for testing and diagnosing Lyme disease when patients are exposed to certain high-risk behavior. For example, the patient in the case report presented with multiple episodes of urticaria without a history of the traditional EM rash. The nerve pain he experienced alerted the dermatologist that Lyme titers were necessary. The delayed diagnosis for this patient increased the chances of progressing to chronic Lyme disease with complication in the treatment course. The drug of choice for localized disease is doxycycline, while other stages might require IV preparations [1]. Multiple studies have shown that delayed treatment lowers the chance of a full recovery, manifesting in prolonged musculoskeletal and neurologic sequela [4]. Along with early detection, a vital step is patient education especially those living or working in grassy, bushy areas. Encouraging protective clothing and applying insect replants containing N, N-diethyl-meta-toluamide (DEET) are preventative measures patients can take [5]. It is imperative to continue researching and discovering dermatologic presentations for Lyme disease so that patients can receive appropriate treatment. Pertaining to the patient in this case, the occupational history combined with cutaneous lesions and neuropathic pain cued the physician that an underlying infectious process could be the culprit. We hope to promote continuing research into Lyme disease-associated skin conditions and to encourage physicians to look beyond the “traditional” lesions.

## Conclusions

Lyme disease can be a devastating condition, which often requires extensive treatment if diagnosis is delayed. For this reason, it is important that physicians be cognizant of atypical presentations. This patient's wide-spread urticaria was the initial presentation of Lyme disease. His occupation of navigating grasslands put him at increased risk, even though the *Ixodes* tick is not rampant in South Florida. Regardless of the region, patients with unorthodox skin eruptions, who participate in high-risk behaviors should be screened and properly treated to avoid long-term issues.
